# Feasibility and Outcomes of a Robotic Retroperitoneal Approach to Para‐Aortic Lymphadenectomy in Intermediate‐ and High‐Risk Endometrial Cancer: A Prospective Study

**DOI:** 10.1002/rcs.70148

**Published:** 2026-03-20

**Authors:** Michiko Kubo‐Kaneda, Kenta Yoshida, Saki Kotaka, Asumi Okumura, Tsuyoshi Mastumoto, Kota Okamoto, Eiji Kondo

**Affiliations:** ^1^ Department of Obstetrics and Gynecology Mie University School of Medicine Tsu Japan

**Keywords:** endometrial cancer, lymph node excision, quality of life, retroperitoneal, robotic surgical procedures

## Abstract

**Background:**

This prospective feasibility study evaluated the safety and efficacy of robotic retroperitoneal para‐aortic lymphadenectomy in patients with intermediate‐to high‐risk endometrial cancer.

**Methods:**

Patients with endometrial cancer who underwent robotic surgical staging at Mie University Hospital between October 2021 and May 2025 were prospectively enroled and analysed following ethics approval. Patient clinical data, intraoperative parameters, and postoperative quality of life (QOL) were collected according to a predefined protocol.

**Results:**

The study included 18 patients with a median operative time of 411.0 min, median blood loss of 51.0 mL, and 25.5 and 24.5 resected pelvic and para‐aortic lymph nodes, respectively. Para‐aortic lymph node metastasis was observed in one case, and postoperative complications in two. QOL returned to baseline within 28 days postoperatively.

**Conclusions:**

The robotic retroperitoneal approach to para‐aortic lymphadenectomy is a feasible and safe staging procedure for intermediate‐to high‐risk endometrial cancer, with rapid postoperative QOL recovery.

**Trial Registration:**

This study was registered in the Japan Registry of Clinical Trials (jRCT1042210047).

## 1 Introduction

Endometrial cancer is one of the most common gynaecological cancers, with an increasing incidence in recent years likely due to changes in diet and lifestyle. In 2025, approximately 420,368 women worldwide were diagnosed with endometrial cancer, while 97,723 women died of this disease [1]. Most cases occur in women aged 50 years and older, especially those with obesity or metabolic conditions such as diabetes. Although advances in treatment have contributed to a high 5‐year overall survival rate of approximately 81.1% [2], surgery remains the standard treatment for endometrial cancer. Total hysterectomy and bilateral salpingo‐oophorectomy are performed for low‐risk endometrial cancers. Meanwhile, staging via pelvic and para‐aortic lymph node dissection or biopsy is required to determine the stage and obtain histological information for adjuvant therapy for intermediate‐to high‐risk endometrial cancer.

Advancements in endoscopic technology have led to an increasing number of minimally invasive surgeries (MIS), including laparoscopic and robot‐assisted surgeries. MIS is considered more effective than open surgery in terms of perioperative outcomes, particularly in patients with endometrial cancer [3]. The rate of MIS performed for endometrial cancer at the National Comprehensive Cancer Network centres was approximately 80% during 2013–2014 [4]. Similarly, in Japan, the proportion of MIS for endometrial cancer at high‐volume centres increased from 24.8% in 2015 to 41.0% in 2019 [5]. In Japan, laparoscopic surgery for patients with stage 1 endometrial cancer has been covered by insurance since 2012, whereas robotic surgery has been covered since 2018. Laparoscopic para‐aortic lymphadenectomy has been covered by national insurance since 2020, and we have performed the procedure using a retroperitoneal approach. However, robotic surgery for this technique has not yet been approved for insurance coverage.

The da Vinci Surgical System is a robotic platform that offers advantages such as dexterity through multi‐joint instruments, tremor filtration, a stable operating field, high‐definition three‐dimensional visualisation for surgeons, reduced surgeon fatigue, and a shorter learning curve over a conventional approach. These features make the da Vinci Surgical System particularly suitable for this technically demanding procedure, which requires precise dissection. However, reports on retroperitoneal para‐aortic lymphadenectomy using the da Vinci Surgical System for endometrial cancer remain limited worldwide, particularly in Japan.

Pelvic and para‐aortic lymphadenectomy was initially established through open surgery at our institution and subsequently transitioned to a laparoscopic approach using combined retroperitoneal and transperitoneal access. Based on accumulated experience and the demonstrated utility and safety of laparoscopic pelvic and para‐aortic lymphadenectomy, a robotic retroperitoneal approach was developed to address the limitations of conventional techniques, particularly in patients with increased surgical complexity.

Therefore, we aimed to prospectively evaluate the feasibility of a robotic retroperitoneal approach for para‐aortic lymphadenectomy in patients with intermediate‐to high‐risk endometrial cancer to assess its safety and efficacy.

## 2 Materials and Methods

### 2.1 Patients

This prospective feasibility study was approved by the Clinical Research Ethics Review Committee of Mie University Hospital (approval no. H2021‐141; August 5, 2021) and adhered to the standards of the Declaration of Helsinki, as revised in 2001. Written informed consent was obtained from all participants before enrolment in the study. Robotic para‐aortic lymphadenectomy was performed as a self‐funded surgery. Among patients meeting the following inclusion criteria at Mie University Hospital between October 2021 and May 2025, those who provided written informed consent were consecutively enroled (*n* = 18): T1b–T2 endometrioid grade 1 carcinoma or T1a–T2 serous, clear, mucinous, endometrioid carcinoma grade 3 and carcinosarcoma of endometrial cancer who underwent robotic surgical staging, including pelvic and para‐aortic lymphadenectomy.

Patients suspected of having lymph node metastasis or peritoneal dissemination preoperatively, those with severe cerebral aneurysm or glaucoma, those with severe comorbidities that could contraindicate general anaesthesia, and those with active multiple malignancies were excluded. The disease stage was classified in accordance with the 2008 International Federation of Gynaecology and Obstetrics (FIGO) staging system [6]. Endometrial tumour histology was based on the World Health Organisation Committee classification of tumours [7].

### 2.2 Clinical and Pathological Data

Clinical and pathological data were prospectively obtained during the study period from patients' medical records. The clinical data included age, body mass index (BMI), nulliparity, endometrial cancer associated history (e.g., diabetes mellitus, hypertension, and hyperlipidaemia), perioperative complications, operative time, blood loss, length of postoperative stay, lymph node count, lymph node status, prognostic information, and quality of life (QOL) assessed using the EuroQol 5‐dimension 5‐level (EQ‐5D‐5L) [8] visual analogue scale (range, 0–100). The patients completed the EQ‐5D‐5L questionnaire preoperatively and on days 1, 4, 14, and 28 after surgery. All patients underwent contrast‐enhanced computed tomography from the chest to the lower extremities on postoperative days 5–7 to evaluate venous thromboembolism and lymphocele, as well as 3 weeks after surgery. The pathological data included histology, depth of myometrial invasion, presence of lymphovascular involvement, and peritoneal cytology results.

### 2.3 Robotic Surgical Procedure

**FIGURE 1:**
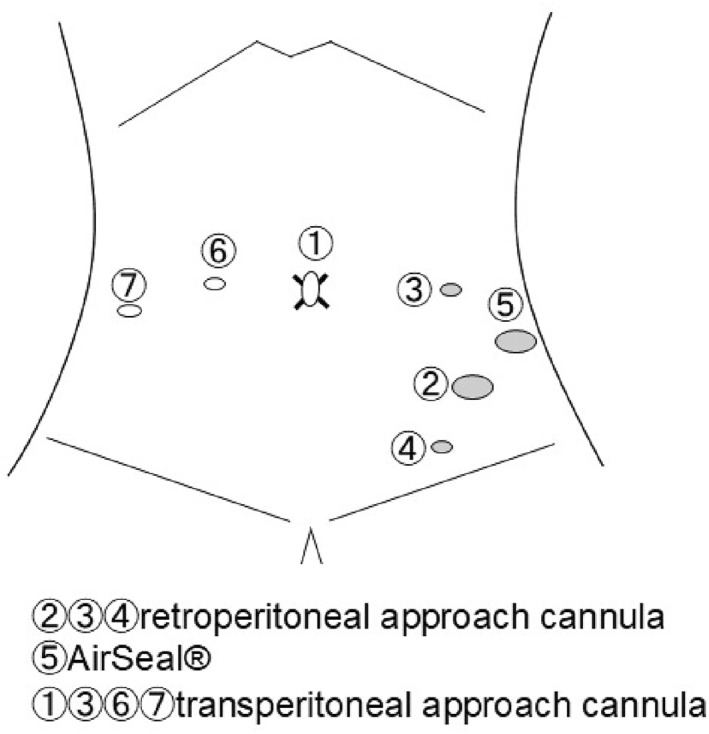
Surgical incision. Cannulas were placed at ②, ③, and ④, AirSeal port was placed at ⑤ for the retroperitoneal approach. Two additional cannulas were placed at ⑥ and ⑦ for the transperitoneal approach.

All procedures were performed by the same lead surgeon. General and epidural anaesthesia were administered in all cases, and surgeries were performed in the lithotomy position using a boot‐type fixator. The pelvic elevation angle was 25°. The lithotomy and head positions were released approximately every 3.5 h. Surgery was performed using a da Vinci Xi (Intuitive Surgical, Sunnyvale, CA, USA). An 8 mm cannula with a Hasson cone (Intuitive Surgical, Sunnyvale, CA, USA) was inserted into the supraumbilical region. An additional 8 mm cannula with a Hasson cone was inserted using the Spacemaker Dissection Balloon: PDB (Medtronic, Minneapolis, MN, USA) opposite the McBurney's point into the retroperitoneal space. Subsequently, 8 mm cannulas were placed in the left upper and lower abdomens, and a 12 mm AirSeal (CONMED, Largo, Florida, USA) port was placed in the left lateral abdomen between points ② and ③, as shown in Figure 1. The inferior mesenteric artery and left ureter were identified, and a retroperitoneal para‐aortic lymphadenectomy was performed from the aorta to the level of the left renal vein. Two additional 8 mm cannulas were placed in the right abdomen, as shown in Figure 1, and a transperitoneal pelvic lymphadenectomy and a modified radical hysterectomy were performed. The following measures were taken in all patients to minimise the risk of tumour spillage: cervical suturing was performed preoperatively; intraoperatively, the fallopian tubes were clipped, and the surgical specimen was placed in a retrieval bag; vaginal washings with saline solution were performed before hysterectomy and abdominopelvic washings were performed after hysterectomy; and tumour specimens were not morcellated or sectioned into smaller pieces.

### 2.4 Statistical Analysis

The primary endpoint of this study was bleeding volume, which was the main parameter for evaluating the safety of robotic surgical staging. The secondary endpoints were incidence of postoperative complications, operative time, number of lymph nodes, QOL, and recurrence rate. Blood loss was selected as the primary endpoint because it is an immediate and objective indicator of surgical safety in para‐aortic lymphadenectomy and may directly affect perioperative morbidity and transfusion requirements. Linear regression analysis was performed to evaluate temporal trends in operative time and BMI according to case order. Continuous variables were analysed using the Mann–Whitney *U* test. Progression‐free survival (PFS) was assessed using Kaplan–Meier survival curves. Continuous variables are reported as median (interquartile range [IQR]). Statistical significance was set at *p* < 0.05. The EQ‐5D‐5L results were analysed, and the significance levels were adjusted using the Bonferroni correction to account for multiple comparisons. Accordingly, the significance level was adjusted to *p* = 0.05/4 = 0.0125. All statistical analyses were conducted using SPSS, version 28.0 (IBM Corp., Armonk, NY, USA).

## 3 Results

**FIGURE 2:**
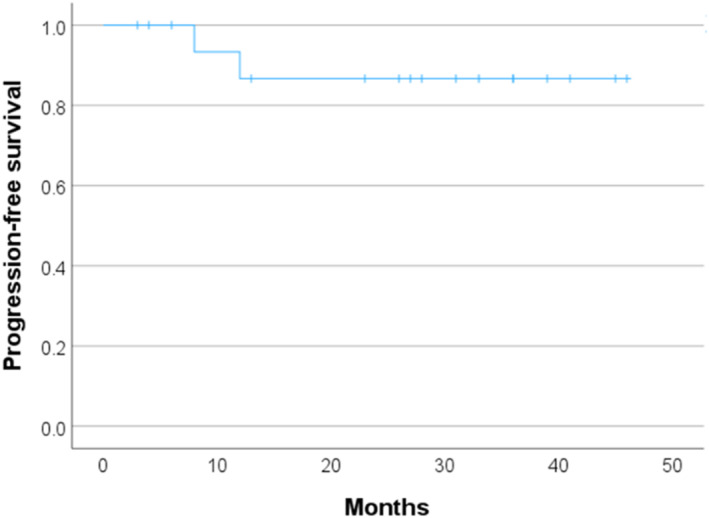
Kaplan–Meier plot of the progression‐free survival. Progression‐free survival of 18 patients was estimated using the Kaplan–Meier method.

This study included 18 patients with a median age of 57.5 (48.5–64.0) years, a median BMI of 24.6 (22.3–27.7), and a median follow‐up time of 29.5 (19.0–38.2) months. The patients' clinical data are shown in Table 1. Approximately 63% of patients had endometrioid carcinoma G1/G2, and 55% had stage T1b–T3. Among these patients, 44.4% had lymphovascular involvement, and 16.7% had positive results on ascitic fluid cytology. Table 2 shows the surgical findings and outcomes. The median operative time was 411.0 (358.0–449.5) min, and the median blood loss was 51.0 (5.7–87.5) mL. The number of resected pelvic and para‐aortic lymph nodes was 25.5 (20.8–27.0) and 24.5 (18.2–31.7), respectively. Para‐aortic lymph node metastasis was observed in one case. Postoperative complications occurred in 2 of 18 patients (11.1%): lymphoedema (Clavien‐Dindo Classification: CDC grade IIIa) and port‐site hernia (CDC grade IIIa), one case each. Linear regression analysis revealed a mild but significant increase in operative time with increasing case order (*β* = + 6.1 min per case, *p* = 0.009). Similarly, BMI significantly increased with increasing case order (*β* = + 0.31 per case, *p* = 0.044). The Kaplan–Meier curves for PFS are shown in Figure 2. Recurrence was observed in two cases, both of which involved bone metastases.

**TABLE 1:** Patient characteristics and pathological findings.

	*n* = 18
Age	57.5 (48.5–64.0)
Body mass index	24.6 (22.3–27.7)
Nulliparous	1.5 (0–2.3)
Complication associated with endometrial cancer^a^	4 (22.2%)
CA125 (U/mL)	17.9 (10.5–23.8)
Histological type	
Endometrioid carcinoma G1/G2	10 (55.5%)
Endometrioid carcinoma G3	4 (22.2%)
Serous carcinoma	2 (11.1%)
Mucinous carcinoma	1 (5.6%)
Carcinosarcoma	1 (5.6%)
Stage	
pT1a	8 (44.4%)
pT1b	8 (44.4%)
pT2	1 (5.6%)
pT3b	1 (5.6%)
Lymphovascular involvement	8 (44.4%)
Positive peritoneal cytology	3 (16.7%)

^a^ Diabetes mellitus, hypertension, and hyperlipidaemia.

**TABLE 2:** Surgical findings and outcomes.

	*n* = 18
Operating time	411.0 (358.0–449.5)
Blood loss	51.0 (5.7–87.5)
Blood transfusion	0 (0%)
Median pelvic lymph nodes	25.5 (20.8–27.0)
Median para‐aortic lymph nodes	24.5 (18.2–31.7)
Metastasis of lymph nodes	1 (5.6%)
Number of operative complications	2 (11.1%)
Chyle or lymphorrhea	1 (5.6%)
Port‐site hernia	1 (5.6%)
Postoperative days	6.0 (5.0–6.2)

**FIGURE 3:**
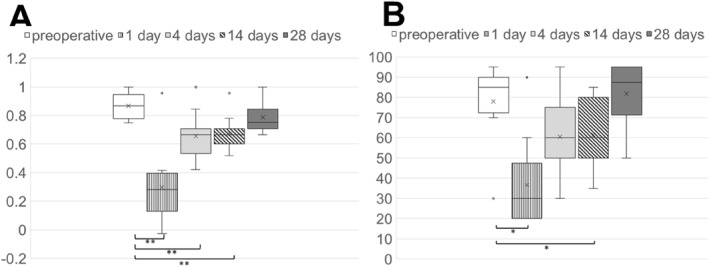
Five levels and visual analogue scale of EQ‐5D‐5L scores. n.s., not significant by Mann–Whitney *U* test. *p < 0.05, **p < 0.01. *p*‐values were adjusted for multiple testing using the Bonferroni correction. The adjusted significance level was *p* < 0.0125.

Results for the five EQ‐5D‐5L levels are shown in Figure 3A. The horizontal line inside each box represents the median value of the data. We applied the Bonferroni correction to adjust for multiple comparisons between the baseline and postoperative values. The median (IQR) QOL scores at baseline (preoperative) and at 1, 4, 14, and 28 days postoperatively were 0.86 (0.78–0.94), 0.28 (0.13–0.39), 0.66 (0.53–0.71), 0.66 (0.60–0.71), and 0.75 (0.71–0.84), respectively. The scores on days 1 (*p* = 0.003), 4 (*p* = 0.004), and 14 (*p* = 0.002) were significantly lower than those at baseline; no significant difference was observed between baseline scores and those at 28 days (*p* = 0.116). The EQ‐5D‐5 L visual findings are shown in Figure 3B. The median (IQR) QOL scores at baseline (preoperative) and at 1, 4, 14, and 28 days postoperatively were 85.0 (72.5–90.0), 30.0 (20.0–47.5), 60.0 (50.0–75.0), 60.0 (50.0–80.0), and 87.5 (71.2–95.0), respectively. The scores on days 1 (*p* = 0.028) and 14 (*p* = 0.017) were lower than those at baseline. No significant difference was observed between baseline scores and those at 4 days (*p* = 0.115), whereas scores at 28 days (*p* = 0.524) were similar to those at baseline.

## 4 Discussion

This prospective feasibility study demonstrated that a robotic retroperitoneal approach to para‐aortic lymphadenectomy is safe and effective for patients with intermediate‐to high‐risk endometrial cancer, as evidenced by minimal blood loss, acceptable operative times, adequate lymph node resection, and a low rate of postoperative complications.

Although operative time showed a mild increase over successive cases, this trend is likely attributable to increasing surgical complexity rather than a negative learning curve. Notably, BMI increased significantly with case order, suggesting that patients with greater surgical difficulty were treated more frequently in later cases. Collectively, these findings indicate that the observed longer operative times are due to a shift towards more challenging cases rather than technical inefficiency.

Analysis of surgical pathological staging in endometrial cancer revealed that the rate of para‐aortic lymph node metastasis was 35.4% in endometrioid carcinoma grade 1 with at least T1b disease and 15.5% in serous carcinoma, even at the T1a stage [9]. These findings indicate that intermediate‐ and high‐risk endometrial cancers are associated with a substantial risk of lymph node metastasis. In this regard, a meta‐analysis of intermediate‐to high‐risk endometrial cancer reported that combined pelvic and para‐aortic lymphadenectomy improves survival outcomes compared with pelvic lymphadenectomy alone [10, 11]. However, this combined approach is associated with a 26% increase in postoperative complications [10], highlighting the need for safe and minimally invasive surgical techniques.

MIS has become the standard treatment for endometrial cancer as it results in reduced blood loss, lower rates of perioperative complications, and shorter hospital stays, while achieving perioperative outcomes, including the number of resected lymph nodes, and oncological outcomes comparable to those of open surgery [12, 13]. Previously, in a study comparing open surgery and laparoscopy, we reported that laparoscopy was associated with reduced blood loss, fewer complications, and shorter hospital stay [14]. However, with the introduction of robotic surgery, the proportion of robotic procedures performed has increased, accounting for 72% of MIS for endometrial cancer in the United States [4]. Subsequently, a retrospective review of patients with high‐grade endometrial carcinoma reported that robotic surgery demonstrated oncological outcomes comparable to those of laparoscopic surgery, with no significant differences in postoperative complications or long‐term survival, including 4‐year progression‐free and disease‐specific survival [15]. In contrast, a systematic review and network meta‐analysis of patients with severe obesity (BMI ≥ 40 kg/m^2^) and endometrial cancer reported that robotic surgery achieved superior outcomes in terms of complete lymph node staging and a significantly lower risk of intraoperative complications compared with open and laparoscopic surgery [16]. Obesity increases perioperative risk due to difficulties in visualising the pelvic organs, as well as an increased risk of anaesthetic and ventilatory complications. In this regard, the abdominal wall‐lifting effect during da Vinci surgery expands the surgical workspace while simultaneously reducing intra‐abdominal and airway pressures, which offers significant benefits, especially for patients with obesity. Therefore, da Vinci surgery is considered useful for endometrial cancer, which frequently occurs in patients with obesity. Accordingly, in a previous study comparing robotic surgery and laparoscopy in patients with obesity (BMI > 30 kg/m^2^) and endometrial cancer who underwent pelvic lymphadenectomy, we reported that robotic surgery resulted in less blood loss and shorter hospital stays compared with laparoscopy [17]. Moreover, our previous study evaluating laparoscopic para‐aortic lymphadenectomy for endometrial cancer at our institution had a median blood loss of 72 mL, a median operative time of 386 min, and a median of 28 and 20 pelvic and para‐aortic lymph nodes resected, respectively. In the current study, the median operative time was 411 min, which tended to be longer but was considered acceptable. Meanwhile, the median number of resected pelvic and para‐aortic lymph nodes was 25.5 and 24.5, respectively. Our results indicate that robotic surgery achieves an adequate lymph node count, as previous reports have suggested that the retrieval of more than 20 lymph nodes is associated with improved prognosis.

Para‐aortic lymphadenectomy for endometrial cancer can be performed using two approaches: transperitoneal and retroperitoneal. Although the retroperitoneal approach is associated with a longer learning curve, its advantages include a reduction in perioperative complications as the intestines and ureters are elevated out of the surgical field [18]. This approach is beneficial in patients with obesity because it avoids the thick mesenteric adipose tissues of the small bowel and colon from the surgical field and permits a reduction in the duration of Trendelenburg positioning. Accordingly, in a prospective randomised open‐label multicenter trial comparing robotic and laparoscopic surgery for para‐aortic lymphadenectomy in endometrial cancer, the retroperitoneal robotic approach was associated with fewer surgical complications [19]. The perioperative complication rate of robotic para‐aortic lymphadenectomy in endometrial cancer, reported to be approximately 20% [20, 21], was 11% in this study, which was within the acceptable range. In addition, the robotic platforms allow comprehensive lymphadenectomy, even in challenging anatomical regions, such as the para‐aortic area, with acceptable complication rates. Moreover, the retroperitoneal approach to para‐aortic lymphadenectomy for endometrial cancer is not widely adopted in Japan. Therefore, the confirmation of its safety in this study may help further develop and refine this surgical procedure.

Oncological outcomes of endometrial cancer are comparable between open surgery and MIS. However, reports have indicated that intraoperative tumour spillage, such as uterine perforation caused by manipulators or serosal involvement during MIS [22], may be associated with an increased risk of recurrence. Moreover, the use of a uterine manipulator [23] is associated with worse oncological outcomes in patients with endometrial cancer undergoing MIS, emphasising the importance of adequate measures to prevent tumour dissemination. In this study, two cases of recurrence were observed despite the implementation of sufficient measures for preventing tumour spillage; however, both were distant metastases, suggesting that tumour spillage was unlikely to be the cause.

Laparoscopy results in more favourable outcomes compared with open and vaginal approaches for endometrial cancer [24]. Additionally, QOL recovery after total robotic hysterectomy for gynaecologic cancers occurs within approximately 4 weeks [25]. In this study, QOL was transiently reduced in the early postoperative period, but returned to baseline by 28 days. This rapid recovery is particularly relevant for patients with endometrial cancer undergoing para‐aortic lymphadenectomy and supports the use of robotic approaches.

Despite the positive outcomes, the present study has certain limitations. First, this was a single‐centre study with a relatively small sample size. Therefore, further multicenter randomised studies are required to clarify the effect of robotic surgery on perioperative outcomes and long‐term survival. Second, sentinel lymph node biopsies were not performed. Given that the utility of sentinel lymph node biopsy in intermediate‐to high‐risk endometrial cancer has also been reported [26], it is expected to play an increasingly important role in the future. Third, the follow‐up period was relatively short, and only a few progression or recurrence events occurred. Thus, the oncological outcomes reported here are preliminary and require confirmation in studies with longer follow‐up.

## 5 Conclusion

The robotic retroperitoneal approach to para‐aortic lymphadenectomy for intermediate‐to high‐risk endometrial cancer is feasible and safe, providing excellent operative and short‐term quality‐of‐life outcomes. These findings support the expanded use of robot‐assisted systems in complex gynaecologic cancer surgeries, which are expected to facilitate the broader adoption of minimally invasive techniques.

## Author Contributions

Conception and design of the study: Michiko Kubo‐Kaneda and Eiji Kondo. Data analysis and interpretation: Michiko Kubo‐Kaneda. Collection and assembly of data: Michiko Kubo‐Kaneda and Eiji Kondo. Drafting of the article: Michiko Kubo‐Kaneda and Eiji Kondo. Critical revision of the article for important intellectual content: Michiko Kubo‐Kaneda. Final approval of the article: Eiji Kondo.

## Funding

The authors have nothing to report.

## Ethics Statement

This study was approved by the Clinical Research Ethics Review Committee of Mie University Hospital (approval no. H2021‐141; August 5, 2021).

## Consent

Written informed consent was obtained from all participants before enrolment in the study.

## Conflicts of Interest

E.K. received payment for lectures from Intuitive Surgical Inc. The other authors have no relevant financial or non‐financial interests to disclose.

## Data Availability

Data are available from the authors upon reasonable request.
